# 

**Published:** 1881-01

**Authors:** 


					﻿JANUARY, 1881.
TWENTY-EIGHTH YEAR.
HALL'S
JOURNAL
OF
HEALTH
E. H. GIBBS, A.M., M.D., Editor,
No. 141 EIGHTH STREET (Near Broadway), NEW YORK.
TERMS : $2 a Year, or $1 for Six Months. Single nnmher, 20 cts.
				

